# Patterns of habitual physical activity in children and adolescents with cerebral palsy (GMFCS I-II)

**DOI:** 10.3389/fspor.2026.1896196

**Published:** 2026-07-31

**Authors:** Marte Fossflaten Tørring, Karin Roeleveld, Stian Lydersen, Ellen Marie Bardal, Siri Merete Brændvik

**Affiliations:** 1 Department of Neuromedicine and Movement Science, Faculty of Medicine and Health Sciences, Norwegian University of Science and Technology, NTNU, Trondheim, Norway; 2 Physiotherapy Unit, Trondheim Municipal, Trondheim, Norway; 3 Regional Centre for Child and Youth - Mental Health and Child Welfare, Department of Mental Health, Faculty of Medicine and Health Sciences, Norwegian University of Science and Technology, NTNU, Trondheim, Norway; 4 Clinic of Rehabilitation, St Olavs Hospital, Trondheim University Hospital, Trondheim, Norway

**Keywords:** activity patterns, activity types, cerebral palsy, habitual physical activity, human activity recognition

## Abstract

**Background:**

To provide tailored recommendations and interventions for physical activity in children with cerebral palsy, it is important to gain insight not only into the amount and intensity, but also into the temporal pattern of activity. This descriptive study describes postures, bouts, transitions, timepoints, frequency, and type of activity in ambulatory children with Cerebral Palsy (CP) and compare these patterns with typically developing (TD) peers.

**Methods:**

Twenty-three children with CP [GMFCS I-II, 11 males, mean age [range] 9 years [7–16]] and 23 TD peers [9 male, mean age[range] 10 years [7–15]] wore two accelerometers for seven consecutive days. A validated human activity recognition model classified following activities; lying, sitting, standing, walking, running, cycling and jumping based on raw acceleration data.

**Results:**

Both groups accumulated about five hours of daily physical activity through active postures and locomotion, predominantly through standing (∼200 min) and walking (∼100 min), and short periods of running (∼10 min), cycling (<1 min) and jumping (1–2 min). The accumulated activity was ∼8% for CP and 10,5% higher for TD on weekdays than weekends. Children with CP were on average ∼10 to 17 min less active and had ∼15% less active-sedentary-transitions compared to TD, but without statistical significance. During weekdays, all children engaged in standing bouts of up to 10 min, walking bouts of up to two minutes and running bouts of up to 24 s. During weekends, the proportion of children performing different bout lengths decreased as bout length increased.

**Conclusion:**

Ambulatory children with CP, predominantly classified as GMFCS I, demonstrated habitual physical activity patterns, with no statistically significant difference from TD peers. Additionally, there was no statistically significant difference between weekdays and weekends for either group. Substantial within-group variation highlights the heterogeneity of habitual physical activity patterns in children. Furthermore, given small sample size and low statistical power, these findings should be regarded as descriptive and hypothesis-generating.

## Introduction

Physical activity is a vital component for optimal physical, emotional, and psychosocial development for all children. Children and adolescents with cerebral palsy (CP) are reported to be less physically active than typically developing (TD) peers. Studies show that only 13%–25% (1, 2) of ambulatory children with CP meet the daily recommendations for physical activity, which is fewer than their TD peers (3, 4). Furthermore, trajectories show a decline in both amount and intensity of physical activity from as early as three years and up to 12 years of age across all levels of the Gross Motor Functional Classification system (GMFCS) (5). The observed reduction in activity levels may have negative consequences for general health and social participation (6–8). This raises an important question of how habitual physical activity can be increased and better regulated in children with CP.

One step towards answering this question is to get deeper insight into habitual physical activity (HPA) for children and adolescents with CP. To date most research on this has focused on total accumulated time in physical activity and different activity intensities (1, 3, 4, 9, 10). While this work has established important foundations, emerging evidence suggests that patterns of HPA may provide additional information about how HPA may be accumulated and regulated (11, 12). A recent Delphi study by Ridger et al. (13) recommends that HPA patterns should be defined as; temporal structures of physical activity and sedentary behaviors accumulated over a specific time period. Furthermore, Ridger et al. (13), and Gomes et al. (14), argue that patterns of HPA should encompass more than intensity (14), including posture, bouts, transitions, timepoints, frequency, and type of activity. The authors emphasize that understanding HPA patterns is essential for tailoring physical activity recommendations. Consequently, a comprehensive approach that integrates all dimensions of HPA to identify meaningful patterns in children with CP is needed.

Advancements in objective measurements of physical activity, particularly the use of raw acceleration signals and machine-learning-based Human Activity Recognition (HAR) models (15) offer new opportunities to address this need, including in pediatric populations (16). These methods allow for detailed, population specific analyses of postures, activities and the temporal structures of movements. Applying these approaches to describe physical activity in children with CP can generate more nuanced and potentially more actionable insights into their HPA patterns, possibly strengthening both research and clinical decision-making.

Therefore, the present study aims to describe daytime patterns of HPA on both weekdays and weekends in Norwegian ambulatory children with CP and compare these patterns with TD peers.

## Methods

### Study design and participants

In this descriptive and explorative cross-sectional study, we included data from 23 children with CP and 23 TD peers. Data for the CP group were obtained from the baseline measurements of an international multicenter randomized controlled trial (RCT) conducted between 2015 and 2021 (ClinicalTrials.gov registration nr: NCT02546999) (17). The present analyses include participants between 7 and 16 years from the Norwegian cohort. Recruitment was conducted in three out of four health regions in Norway. Detailed study procedures and inclusion criteria are described in the original RCT publication (17). Data for TD peers was obtained from a cross-sectional study, between 2016 and 2017, investigating HPA in children and adolescents, recruited through a local primary and junior high school. Although data were collected at different time points, identical equipment, standardized protocols, and consistent procedures were used in both studies.

Written informed consent was obtained from all legal guardians and participating children. The collection and use of CP data was approved by the Regional Ethical Committee for Medical and Health science (no. 2016/707/REK North), while the TD data was approved by the Norwegian Centre for Research Data (no. 50683).

### Inclusion criteria

Participants were included in the present analysis if they had at least two weekdays and one weekend day with complete activity data, in accordance with the minimum recommended number of measurement days (18, 19). A complete day was defined as having 24 h of accelerometer data. Days with 24 h of data were excluded if they were classified as non-wear days. A day was considered non-wear if it included more than 18 h of lying, more than 18 h of sitting, or a combined total of ≥23 h and 24 min of sitting and lying. In addition, the first measurement day was excluded for all participants, because this was a test day, and to reduce risk of Hawthorne effects (20).

Based on these criteria, a total of 23 participants with CP, classified with GMFCS level I and II, and 23 age- and gender-matched TD controls were included in the final analysis. Participants with CP were matched with TD participants using a hierarchical matching procedure, based on age, gender, and season of assessment. When an exact match across all variables was not possible, matching priority was given sequentially to age, gender, and season. Participant descriptives are presented in Table 1. In total, 17 had exact matches, two did not match on age (one year difference), four did not match on gender, and four did not match on season of the year. All data was collected during the school year.

**Table 1:** Descriptives of participants.

Descriptives	Cerebral Palsy	Typically developing
Participants	*N* = 23	*N* = 23
Gender M/F	(11/12)	(9/14)
Age (years, months) Mean with (SD) [Min-Max]	9y, 10 m (2y, 2 m) [7–16]	10y 0 m (2y. 1 m) [7–15]
Height (cm) Mean with (SD) [Min-Max]	138.3 (17.5) [107.5–177.4]	146.8 (12.2) [122–173]
Body Mass (kg) Mean with (SD) [Min-Max]	34.8 (11.7) [20–58]	42.8 (12.2) [26.3–76.8]
GMFCS (I/II)	(18/5)	NA

N, number of participants; M, male, F, female, y, years, m, months, cm, centimetre, kg, kilo gram, NA, not applicable. GMFCS, gross motor function classification system.

### Procedure, equipment and data processing

Habitual physical activity was assessed using accelerometry. All participants were instructed to wear two Axivity AX3 accelerometers (Axivity Ltd, Newcastle, UK) for seven consecutive days, including five weekdays and two weekend days. One accelerometer was attached to the mid-thigh (CP; on least affected side), and one to the lower back at L3 level. Data was sampled at 100 Hz for TD and 200 Hz for CP. For additional details see Brændvik et al. (17). Accelerometer signals were down sampled to 50 Hz, synchronized, and segmented into three seconds windows. Activity classification was performed using a validated Extreme Gradient Boosting (XGBoost) machine-learning model (NTNU-HARChildren), with overall accuracy of 94% in standardized activities and 81% in free play. The model classified the following activities and postures: lying, sitting, standing, walking, running, jumping, and cycling (21).

To describe daytime patterns of HPA, lying during nighttime was excluded and shuffling was relabeled to either standing or walking. The exclusion and relabeling procedures are described in Supplementary Material. Thereafter, the following outcome variables were calculated; 1) Time in different types of activities (standing, walking, running, jumping and cycling) and postures (sitting and daytime lying), 2) Bout lengths and frequency for standing, walking and running, 3) Number of transitions between activities and sedentary postures. All outcome variables were calculated as averages per day for weekdays and weekend days separately. Standing was included as an activity, since this posture often is part of walking, turning and shuffling in children, and has higher energy expenditure than sitting and lying (22).

For pattern analysis, a bout was defined as minimum nine seconds of the same activity. Very short durations are likely to represent brief change in movement embedded within other activities rather than true change in activity behaviour. Therefore, slightly longer durations were required to define a new activity bout. Further details on bout definitions and post-processing steps are provided in Supplementary Material.

### Statistical analysis

Descriptive statistics are reported as mean (standard deviation, SD) and range for continuous variables and counts for categorical variables. Linear mixed-effects models were used, with one physical activity measure at a time as the dependent variable. Group (CP vs. TD), time (weekday vs. weekend), and their interaction were included as covariates, and participant as random effect. Normality of residuals was assessed by visual inspection of Q-Q plots. We used bootstrapping with the BCa (bias corrected and accelerated) option, creating 5000 bootstrap samples. Formal normality tests were not used due to their known limitations in small samples (23), and bootstrapping was therefore considered a more robust approach for statistical inference.

Statistical significance was set at *p* < 0.05. To account for multiple testing, the Benjamini–Hochberg procedure was applied to preserve the false discovery rate. This procedure was implemented in R (R Foundation for Statistical Computing, Vienna, Austria), while all other analyses were conducted using Stata Statistical Software version 18 (StataCorp LLC, TX, USA).

### Data availability and missing data

The average days of complete data per participant was 3.6 weekdays in both the CP and TD groups (range: 3–4 days). For weekend days, participants in the CP group contributed a mean of 1.9 days (range: 1–2), while participants in the TD group contributed a mean of 1.7 days (range: 0–2). This resulted in a total of 86 weekday observations and 43 weekend observations in the CP group, and 80 weekday observations and 39 weekend observations in the TD group. In descriptive analyses, missing data was handled using available cases. In the linear mixed-effects models, participants with partially missing data were included in the analyses.

Figures in the Results section are based on total numbers of observations. Figure 2 presents the percentage of participants who accumulated at least one valid bout of the specified activity across their complete observations. Figure 3 is derived from Figure 2 and includes only observations from participants who demonstrated valid bouts, thereby representing a subset of the total study sample. The number of observations contributing to Figure 3, as well as mean frequency values for full study population, is reported in Supplementary Table S2 in the Supplementary Material.

## Results

Time in different activities and postures, bout lengths and frequency, and number of transitions are presented with corresponding results for weekdays and weekends in the figures and Table 2. The mean time in bed for both groups of children was about 9.5 h [range: 4.0–12.3 h], resulting in about 60% awake time during a 24-hour period.

**Table 2:** Time in different habitual physical activities. .

	Descriptive statistics						Estimates based on linear mixed models				
Activity	N	CP		N	TD		Differences (with uncorrected P-values)				
	N (n)	Weekdays	Weekend	N (n)	Weekdays	Weekend	CP vs TD (weekdays)	CP vs TD (weekends)	CP (weekdays vs weekends)	TD (weekdays vs weekends)	Interaction group × time
	wk/wknd	Mean (SD) [min-max]	Mean (SD) [min-max]	wk/wknd	Mean (SD) [min-max]	Mean (SD) [min-max]	Estimate (95% CI) *P*-value	Estimate (95% CI) *P*-value	Estimate (95% CI) *P*-value	Estimate (95% CI) *P*-value	Estimate (95% CI) *P*-value
Standing minutes	23/23 (23/23)	194.9 (70.0) [50.6–523.5]	187.4 (71.6) [54.2–389.1]	23/21 (23/21)	201.0 (60.2) [43.0–538.4]	184.3 (57.9) [44.7–310.7]	−6.8 (−27.5 to 14.0) *p* = 0.519	1.1 (−28.3 to 30.6) *p* = 0.940	−10.2 (−37.4 to 17.1) *p* = 0.465	−18.0 (−36.1 to 0.4) *P* = 0.056	7.9 (−25.3 to 41.1) *p* = 0.639
Walking minutes	23/23 (23/23)	92.0 (41.9) [9.4–276.7]	79.4 (33.1) [18.4–156.4]	23/21 (23/21)	102.1 (35.6) [7.85–165.5]	88.0 (47.0) [16.8–228.5]	−10.3 (−24.6 to 4.0) *p* = 0.158	−8.2 (−27.6 to 11.1) *p* = 0.403	−14.4 (−28.0 to −0.8) *p* = 0.037	−16.5 (−33.2 to 0.3) *p* = 0.054	2.1 (−19.5 to 23.6) *p* = 0.852
Running minutes	23/23 (23/22)	8.3 (8.5) [0.0–35.0]	5.6 (6.0) [0.0–25.3]	23/21 (23/21)	10.5 (9.3) [0.0–42.4]	7.3 (9.5) [0.0–36.4]	−2.3 (−5.6 to 1.1) *p* = 0.184	−2.1 (−5.8 to 1.6) *p* = 0.259	−3.2 (−6.6 to 0.2) *p* = 0.062	−3.3 (−6.4 to −0.2) *p* = 0.034	0.1 (−4.4 to 4.7) *p* = 0.956
Jumping minutes	23/23 (20/20)	2.0 (6.2) [0.0–34.7]	1.3 (2.9) [0.0–16.7]	23/21 (22/18)	0.8 (1.9) [0.0–13.0]	1.6 (3.6) [0–15.5]	1.1 (−0.4 to 2.6) *p* = 0.151	−0.3 (−1.7 to 1.0) *p* = 0.642	−0.8 (−2.4 to 0.9) *p* = 0.370	0.7 (−0.2 to 1.5) *p* = 0.131	−1.4 (−3.3 to 0.5) *p* = 0.136
Cycling minutes	23/23 (9/5)	1.1 (4.0) [0.0–22.4]	0.3 (1.6) [0.0–10.7]	23/21 (22/18)	0.2 (0.7) [0.0–3.8]	0.2 (0.8) [0.0–4.6]	0.92 (0.1 to 1.8) *p* = 0.030	0.1 (- 0.5 to 0.6) *p* = 0.836	−0.9 (−1.8 to 0.0) *p* = 0.042	−0.0 (−0.3 to 0.2) *p* = 0.881	−0.9 (−1.8 to 0.0) *p* = 0.057
Total active time minutes	23/23	298.4 (99.4) [78.2–534.9]	274.0 (104.4) [72.9–564.3]	23/21	314.6 (71.2) [97.5–575]	281.4 (95.0) [61.7–479.3]	−17.3 (−46.4 to 11.8) *p* = 0.243	−9.7 (−57.6 to 38.3) *p* = 0.693	−29.4 (−71.1 to 12.4) *p* = 0.168	−37.1 (−70.0 to −4.1) *p* = 0.027	7.7 (−45.5 to 60.8) *p* = 0.777
Active-Sedentary transitions	23/23	231.0 (203.8) [37–1,744]	241.3 (128.3) [42–661]	23/21	270.8 (213.5) [72–1,889]	273.8 (209.3) [50–1,032]	−40.7 (−108.3 to 26.8) *p* = 0.237	−51.2 (−138.1 to 35.7) *p* = 0.248	1.1 (−49.3 to 51.5) *p* = 0.966	−11.6 (−44.28 to 67.4) *p* = 0.685	−10.5 (−85.6 to 64.7) *p* = 0.785

N, number of participants that are included in the estimate (weekdays/weekends); n, number of participants having the activity in their measurements, rest have 0 min of the activity (weekdays/weekends). CP, cerebral palsy; TD, typically developing; wk, weekdays; wknd, weekends; min, minutes. Total observations (days included) are 86 for CP and 80 for TD on weekdays and 43 and 39 days on weekends.

### Time in different activities and postures

**Figure 1:**
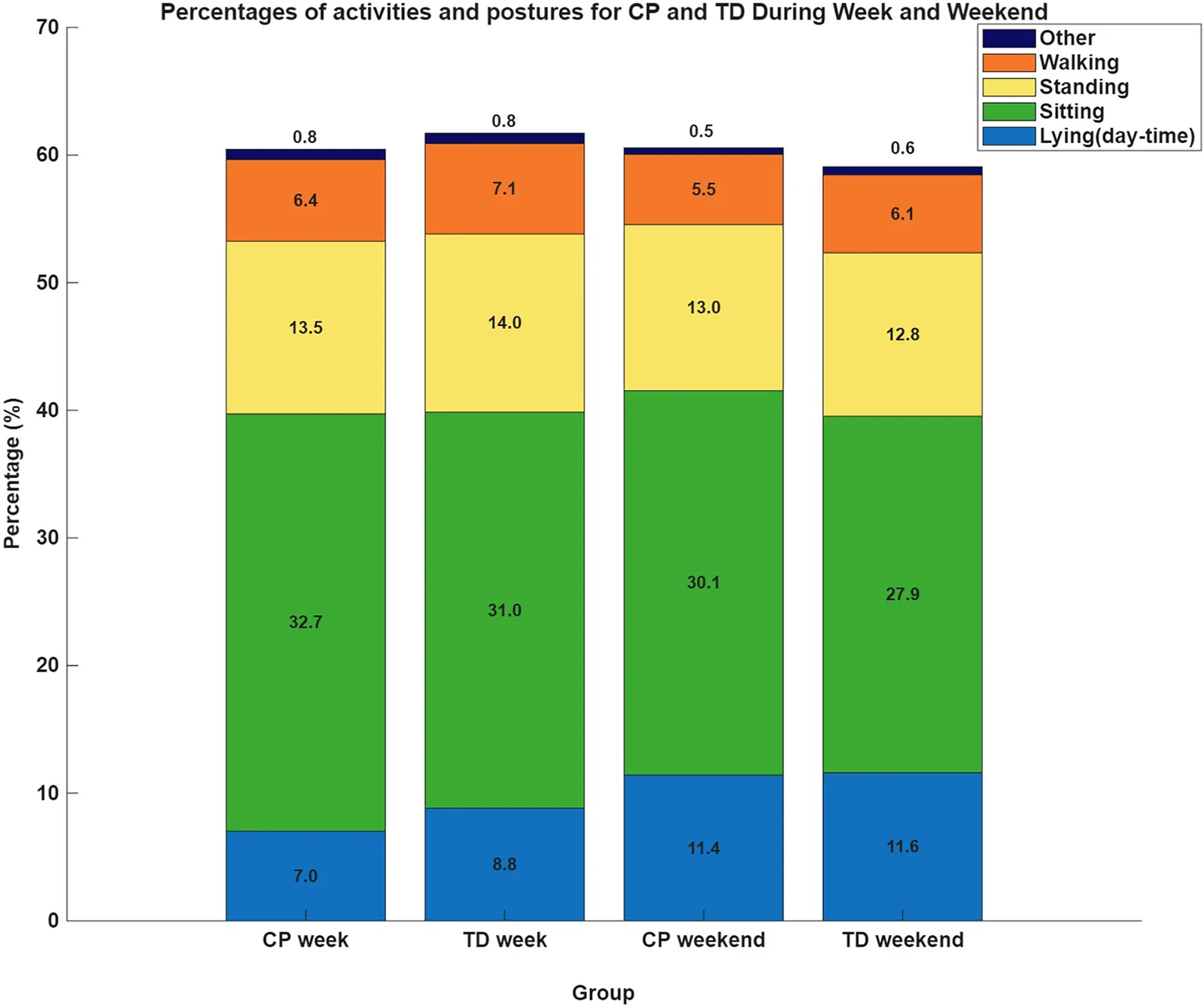
Percentages of awake time activities and postures for CP and TD during week and weekend. Average percentage out of 24 h, across all observations in the four groups, including five different activities. Percentages are provided in the bars. The numbers over the bars represent percentages for ‘Other’ activities: running, jumping and cycling. Groups: CP Week, cerebral palsy on weekdays, TD Week: typically developing children on weekdays; CP Weekend: cerebral palsy on weekends; TD weekend, typically developing on weekends.

Figure 1 shows that the percentage of time spent in different activities and sedentary postures did not substantially differ between children with CP and TD and both groups are slightly less active during weekends than weekdays. Both groups spent about 21% (5 h) of a 24-hour day in physical activity, comprising active postures (standing) and locomotion. Most of this active time (70%) was spent standing (∼3.5 h), followed by walking (∼1.5 h) and limited time spent running, jumping and cycling (∼10 min). Although children with CP spent on average 10 min less time in activity on weekdays and 17 min less on weekends compared to TD children, these differences were not statistically significant. Large variations in total time were observed both within the CP and TD group, with a range from 62 to 575 min in total active time (Table 2). Moreover, all children spent about 7.3 h [2.3–13.1 h] sitting and 2.2 h [0.0–7.2 h] in daytime lying.

As shown in Table 2, some differences between groups and between weekdays and weekends were initially statistically significant. However, after adjusting for multiple comparisons (35 tests) to control the false discovery rate, none of these differences remained statistically significant, with lowest adjusted *p*-value being 0.24.

No significant Group × Time interactions were found across any activity variables, suggesting that differences in habitual physical activity from weekdays to weekends did not differ between children with CP and TD peers.

### Bout length and frequency

**Figure 2:**
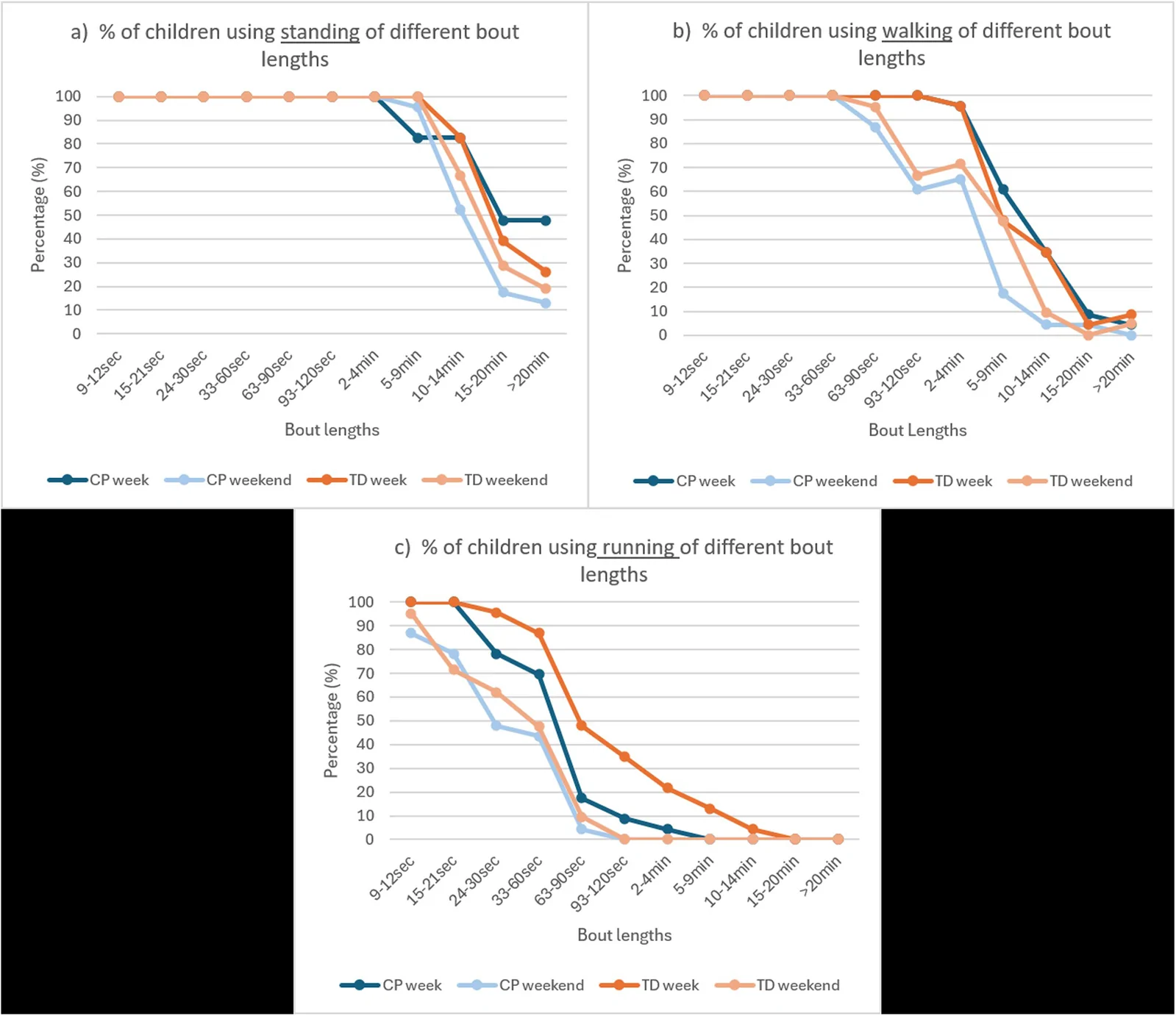
Percentage of children in each group (CP vs TD and weekend vs weekday) using a) standing,b) walking,and c) running across bout lengths. Percentages of children that have at least one instance of the bout interval across their available measurements for the activities standing, walking and running. On both weekdays and weekends. TD Weekend have 2 children with missing data, thus 21 possible children, in all other conditions there are 23 possible children. Groups: CP Week, cerebral palsy on weekdays; CP Weekend, cerebral palsy on weekends; TD Week, typically developing children on weekdays; TD weekend, typically developing on weekends; Bout length, time categories.

Figure 2 shows the percentage of children engaging in different bout lengths of standing (a), walking (b) and running (c) across all observation days. All children in both groups engaged in short activity bouts up to ten minutes for standing, two minutes for walking and 24 s for running. The percentage of children decreased as the bout length increased. Moreover, fewer children engaged in longer bouts on weekends across all three activities and in both groups.

**Figure 3:**
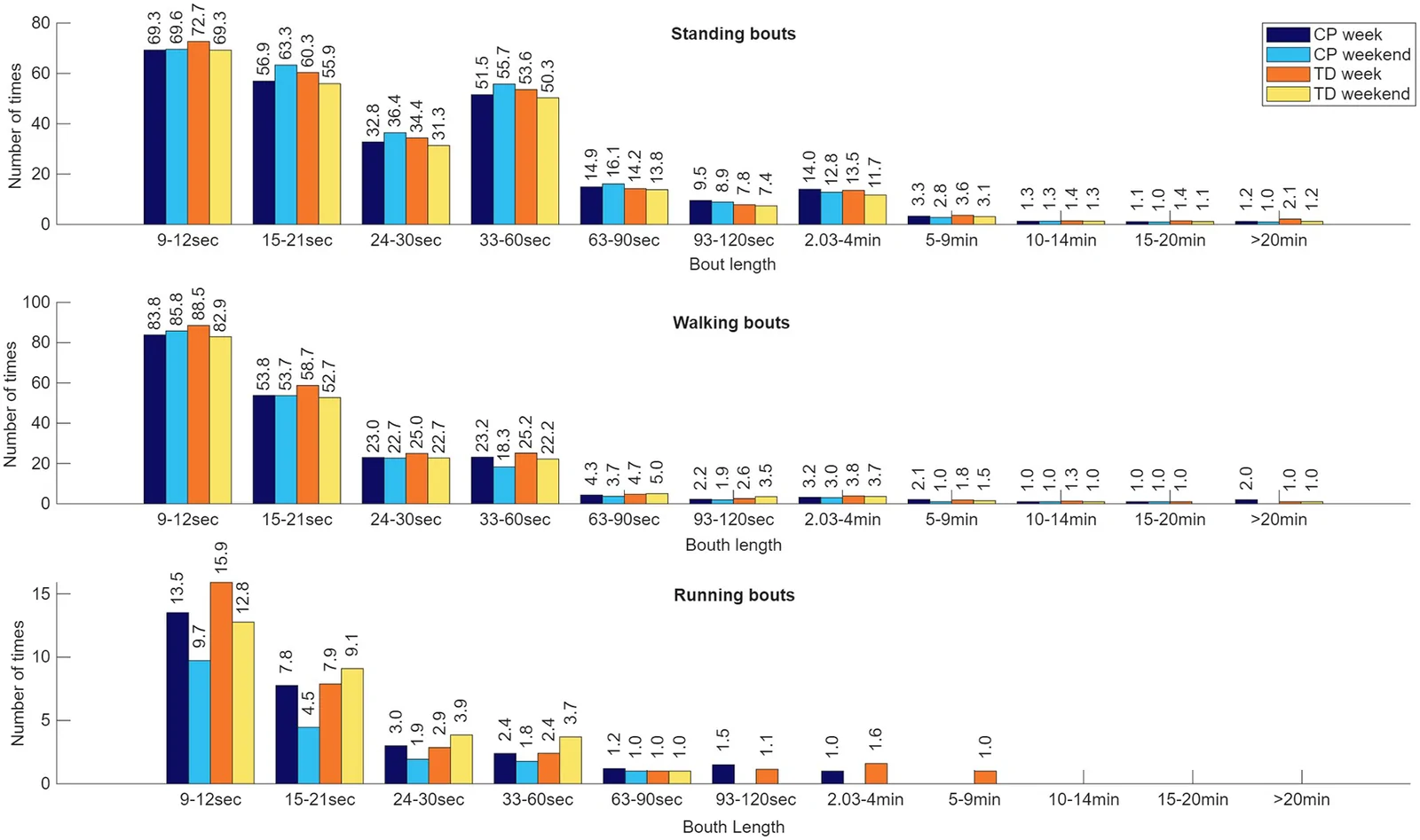
Average frequency in bout lengths for activities standing,walking and running. Average frequency of bout lengths among participants who accumulated bouts, as identified in Figure 2 in the activities standing, walking and running. Only observation days that include the bout length are included in the estimate, for number of observations see Supplementary Table S2 in Supplementary Material. Groups: CP Week, cerebral palsy on weekdays; CP Weekend, cerebral palsy on weekends; TD Week, typically developing children on weekdays; TD weekend, typically developing on weekends; Bouts, Time categories; sec, seconds; min, minutes.

Figure 3 shows the average frequency at which the participants that had the bout lengths in their measurements performed standing, walking and running. The highest frequency can be seen in the shortest (9–12sec) bouts for standing (about 70x/day), walking (about 85x/day) and running (about 10x/day). For all groups the larges reduction in frequency is after the 33–60sec bouts with approximately 35% in standing and 18% in walking. In running bouts, it is a steady decrease from the 9–12sec bouts, with few bouts over 20 s The number of children contributing to the frequency calculations is presented in Supplementary Table S2.

### Transitions between active and sedentary activities

The children in the CP group had on average about 230 transitions between activities and sedentary postures per day. On average, children with CP had about 15% fewer transitions compared with TD peers, but without statistical significance (Table 2). No significant differences in number of transitions between weekdays and weekends were found, for either of the groups.

## Discussion

This study describes the daytime patterns of HPA on weekdays and weekends for ambulatory children with CP and compares these with TD peers. No statistically significant difference in HPA patterns were found between groups, in terms of time in activities, bout lengths, frequencies, and transitions. In time in minutes both groups are less active on weekends than weekdays, and there were no group by time interaction effect. However, the variations in time in activities were large within both groups.

Our results suggest no difference in total activity between children with CP and their TD peers. This contrasts with previous findings of reduced physical activity in children with CP [1, 3–5, 10], most of which evaluated HPA as total accumulated time in different intensities. There are a few studies that evaluate activity patterns by time in different types of activities, however without comparison to TD peers. For example, Shkedy Rabani et al. (24), found that children with CP, GMFCS I and II spent 82.5% of their time lying or sitting, 11.4% standing and 6.1% walking, which is consistent with our findings of approximately 80% lying or sitting, 13% standing and 7% walking or running. Gerber et al. (25) reported a slightly different pattern of 68% lying or sitting, 22% standing and 8% walking or running in GMFCS I- III. Although all three studies, including ours, are comparable by including primary children with GMFCS I and II, Gerber et al. (25), differ methodologically in their use of body acceleration for posture detection, cadence for walking classification, and intensity for running, which may explain the slight differences from our results.

To our knowledge there are few studies that have investigated bout length and frequency when using activity types rather than intensities in children with CP. The study by Shkedy Rabani et al. (24), found a median of 16 s for walking events in GMFCS II. While Ruch et al. (26), found that 95% of walking bouts lasted for eight seconds or less in TD children, while running was five, and jumping four seconds or less. Our findings of mostly short bouts and few children with CP engaging in walking over two minutes and running over one minute, are supported by Gerber et al. (25).

There are no specific recommendations of bout lengths for health benefits in children (27). However, research on physical activity intensities in TD children has reported no significant health benefits associated with extending bout lengths from one to ten minutes, provided that the total accumulated time remained constant (27). Furthermore, short bouts of activity seem to have a positive influence on health outcomes (28). Thereby, it could be hypothesized that shorter bouts of activities, like we found in our dataset, have positive health benefits and should be encouraged. Nevertheless, further research is needed to determine meaningful bout lengths tailored to specific population groups, and how these align with desired health outcomes.

The exact health effects of breaks in sedentary postures are uncertain, but it is hypothesized to have a general positive effect on health outcomes for children (27–29). In our study, breaks in sedentary postures are reflected by the number of transitions, where the children with CP had ∼15% less transitions than TD peers, however not significantly different from TD. The variations in daily transitions in our dataset were considerable with a range from 37 to 1744 in the CP group and 50–1889 in the TD group. Previous studies have also examined transitions in children with CP (24), although differences in definitions and analytical methods currently limit meaningful comparisons across studies. Furthermore, in our study, the use of shorter window sizes to identify activities may have contributed to a higher number of detected transitions compared to previous studies. To our knowledge, there are no studies evaluating health effects of transitions in children with CP, and thereby more research is needed to conclude if the average difference between the CP and TD group of ∼35 daily transitions could be clinically relevant.

Both children with CP and TD peers were more active, in average minutes on weekdays than weekends. However, these differences were not statistically significant after adjustment for multiple testing. Considerable within-group variability was observed in both groups across weekdays and weekends, with total active minutes ranging from 62 to 575 min per day. The general patterns of more total activity on weekdays compared to weekends is consistent with previous findings in both TD children and children with CP (2, 30).

The absence of significant Group × Time interactions indicates that weekday–weekend differences in HPA were not statistically different between groups. One possible explanation is that the structured nature of school days, including recess, physical education, active transportation, and organized activities, promotes physical activity in all children, including children with disabilities (31). It is also possible that the absence of group differences is partly related to the predominance of children classified as GMFCS level I in our sample, as participation in leisure-time activities is generally greater among children with higher functional abilities (32). However, the present study did not collect contextual information on activity participation, and the factors underlying these weekday–weekend differences remain speculative. The substantial within-group variability further supports cautious interpretation, as individual differences may mask potential interaction effects.

### Considerations and limitations

Some considerations to this study must be made. A key strength is the use of a HAR model validated on both children with CP (GMFCS I–II) and TD peers, demonstrating high overall accuracy (94% in standardized activities; 81% in free play). More specifically, for activities typically represented by longer, more continuous bouts, the model shows excellent accuracy, identifying lying and sitting with 99% accuracy, and standing and walking with over 91% accuracy (21). Although accuracy decreases for very short and intermittent activities, misclassifications occur mainly between closely related categories. However, the distinction between sedentary postures and active behaviors remains robust. Cycling had lower classification accuracy (65%) (21). Thereby, with the limited time in cycling in the present study (nine children with CP; ∼1 min average), the cycling estimates warrant cautious interpretation.

Generalizability is somewhat constrained by the predominance of children at GMFCS level I, reflecting a well-functioning subgroup of the CP population. As a result, these children may be more likely to exhibit HPA patterns closer to those observed in TD peers, and the findings may therefore not be representative of children with more severe functional limitations. While the matching procedure strengthened comparability between groups, matching was not complete, with four gender and four season mismatches, corresponding to approximately 17% mismatch in each category, which may introduce some residual heterogeneity and potential bias. However, all data was collected during the school year, reducing variability related to holiday periods or major seasonal changes. The children with CP had lower body mass and height compared to the TD group. Although such differences are consistent with some previous research in children with CP, GMFCS I-II (33), they may nevertheless influence HPA patterns and should therefore be considered when interpreting between-group differences It should also be noted that commonly used anthropometric measures may not fully capture body composition in this population, and may therefore be misleading (34). Additionally, the broad age range may have contributed to variability in activity patterns.

The small sample size (*n* = 23 per group) reduced statistical power, although the stringent inclusion criteria resulted in high-quality, largely complete datasets for both groups. Although physical activity was measured over a 7-day period, which is standard practice, it may not fully capture week-to-week variability or represent a typical week for all participants.

Finally, the absence of contextual data restricts interpretation of underlying determinants of physical activity behavior but does not detract from the study’s ability to describe observed patterns.

### Implication for rehabilitation management and future perspectives

New insight into patterns of HPA in children with CP may contribute to the development of tailored interventions that supports participation in HPA. Fatigue and pain are common complaints among children and adolescents with CP (35, 36), and may contribute to constrain HPA. A better understanding of how activity patterns can be optimized without increasing fatigue or pain is therefore important for both clinical practice and future intervention development.

In this study, children with CP showed, on average, 10–17 min less total activity compared to TD peers. However, no statistically significant differences were observed between groups. Although previous research suggests that short bursts of activity may have positive health effects (28), and that even small increases in daily physical activity can improved health outcomes in youth (37), the current study was not sufficiently powered to determine if these observations are clinically meaningful. Consequently, these findings should be regarded as exploratory and hypothesis-generating rather than conclusive.

The substantial within-group variability further highlights the heterogeneous nature of HPA patterns in both children with CP and TD peers. Larger studies are therefore needed to determine whether differences in HPA patterns between children with CP and TD peers exist and identify patterns that may be associated with improved health outcomes.

As previously discussed, earlier studies have used varying definitions and methods to assess HPA patterns in children, often focusing on total accumulated time in different intensities or weekday variations [1, 3–5, 10]. Furthermore, Gomes et al. (14), concludes that activity patterns in children are operationalized differently across studies. These differences in definitions, measurement equipment, and analytical approaches limit profound comparisons of our findings with most previous studies.

Advances in technology, such as HAR-models, enable more detailed and nuanced measurements of children's HPA patterns. To fully benefit from these developments, greater consensus is needed regarding which aspects of activity patterns are clinically relevant and how these should be defined and reported. Accordingly, future studies on HPA patterns should use transparent and standardized definitions of activity patterns when identifying activity and sedentary patterns associated with positive health outcomes. Furthermore, how such patterns can be promoted without increasing fatigue or other adverse consequences. Such knowledge may provide valuable guidance for clinicians, families, and public health initiatives aimed at supporting healthy HPA patterns in all children.

## Conclusion

Ambulatory children with CP, predominantly classified with GMFCS I, accumulated less activity on average than TD peers and both groups tended to be more active on weekdays than weekends. However, no statistically significant differences in habitual physical activity patterns were detected between groups, nor were any interaction effects observed. Given the small sample size and low statistical power these findings should be regarded as descriptive and hypothesis-generating rather than confirmatory, providing foundation for further exploration of habitual physical activity patterns in children with CP. Additionally, the considerable within-group variation highlights the heterogeneity of habitual physical activity patterns.

## Data Availability

The datasets supporting the findings of this study are available from the corresponding author upon reasonable request.
