# Supplementary material for: Patterns of habitual physical activity in children and adolescents with cerebral palsy (GMFCS I-II)
Source: Front Sports Act Living. 2026 Jul 31;8:1896196. doi: 10.3389/fspor.2026.1896196 (PMC13491053; doi:10.3389/fspor.2026.1896196)
Supplement: Supplementary file 1 [file Supplementaryfile1.pdf]

Supplementary material

Post Processing

Relabelling activities

We relabelled shuffling between walking bouts to walking, in all other scenarios shuffling was labelled as standing. Bending was relabelled to the activity that occurred before the bending. Walking up and down stairs were collapsed with level walking and sitting and standing cycling were collapsed to one activity of cycling. To be considered as cycling, the activity must last for a minimum of 12 seconds/four consecutive bouts of cycling. This precaution is necessary to avoid mislabelling cycling when a child performs cyclic movements for shorter durations.

Bouts

In bouts under one minute, two consecutive 3-second windows of different activity were required to end the bout, for bouts over one minute three consecutive 3-second windows were needed. In all cases, up to 15 % of other activities within a bout was allowed, provided they did not exceed the consecutive bouts limit. Our script started by identifying the first activity each day and then switched activities continuously as they occurred.

Estimated time in bed

Our variable of estimated time in bed (ETB) was calculated as one consecutive bout and gave us estimated sleep time averages between two days. Our script operated under the assumption that participants are in bed at 3 AM, tracing both backwards and forwards in time. If there was more than five minutes of activities other than lying within the next 15 minutes, the period in bed was considered to have ended. Regardless, if the activity was still lying without five minutes of interruption by 7:30 PM and/or 10 AM the ETB was ended. If the day before or after ETB was labelled as non-wear the ETB was not used, as we could not define start or end. To differentiate between the daytime lying (non-sleep) and nighttime lying (ETB) we also calculated ETB for each day, measured between 00:00 and 00:00.

Non-wear

To establish the start and end of wear time, we created a function that identified the first activity that was not lying and sitting at the beginning and the end of the acceleration file. Subsequently, it verified whether there was less than 50% of sitting or lying (non-wear) in the

next 45 minutes/900 epochs to start or end the measurement; otherwise, it proceeded to the next activity and repeated the assessment.

Creation of the non-wear function

In some files, the predicted start and end times did not align with the actual measurement period, defined by when the accelerometers were attached to the participant. The interval between the start of the prediction file and the true start of measurement reflects a pre-recording phase, during which the sensors had been initialized but not yet placed on the subject. During this period, the devices were often stationary (e.g., on a table or in a researcher's pocket), leading the model to classify the activity as sitting or lying.

We identified this interval from our measurements by the following: A grid search was conducted to determine optimal threshold values for identifying and removing non-wear periods at the beginning and end of each file. The search initially explored a wide range of values for window length and threshold percentage (Table S1). For each iteration, the mean absolute error (MAE) was calculated between the manually identified start and end times from the raw acceleration data and the corresponding start and end times obtained using each set of threshold values. In a second, refined search, the optimal criteria were determined to be a 45-minute window (900 epochs) with less than 50% of epochs classified as sitting/lying for a segment to be retained as valid data (Table S1).

Thereby, we created our function that aims to find the correct start and end of each file. The function iterates forward from the start, and backward from the end though each three sec epoch until it reaches one that is not the target code. The function is run twice, once with lying and once with sitting as the target code.

Table S1. Results from grid search to establish optimal threshold values for non-wear removal.

Minutes	Percentage threshold	Mean absolute error
**Test #1**		
20	0.05	1299.45
20	0.2	431.27
20	0.35	189.18
20	0.05	145.73
20	0.75	101.27
30	0.05	4669.09
30	0.2	861.0,
30	0.35	283.91
30	0.50	112.36
30	0.75	80.82
40	0.05	6378.91
40	0.2	3865.55
40	0.35	1458.54
40	0.5	6378.91
40	0.75	102.0
50	0.05	6752.45
50	0.2	4723.18
50	0.35	1885.45
50	0.5	1391.91
50	0.75	102.0
60	0.05	6898.18
60	0.2	4736.73
60	0.35	2020.64
60	0.5	1432.82
60	0.75	118.27
**Test # 2**		
30	0.7	86.27
30	0.8	80.82
35	0.5	112.36
35	0.6	86.27
35	0.7	80.82
35	0.8	102.0
40	0.5	86.27
40	0.6	80.82
40	0.7	98.09
40	0.8	102.0
45	**0.5**	**80.82**

45	0.6	80.82
45	0.7	102.0
45	0.8	102.0

Results from grid search to establish start and end of measurement files. Minutes is the time period it searches, percentage is the threshold of sitting or lying that is allowed in this time period, and mean absolute error is the distance (number of epochs) from manual label of start/end to the new predicted start/end. The highlighted threshold value in red is the one that was used for the calculation of wear time in our study.

Table S2. Average frequency of bout lengths across all participants and across observations

	Children with CP						TD children					
	Weekdays			Weekends			Weekdays			Weekends		
	Standing											
**Bout length**	Mean All (n=86)	Number of observations	Mean from observations	Mean All (n=43)	Number of observations	Mean from observations	Mean All (n=80)	Number of observations	Mean from observations	Mean All (n=39)	Number of observations	Mean from observations
9-12sec	69,3	86	69,3	69,6	43	68,6	72,7	80	72,7	69,3	39	69,3
15-21sec	57,0	86	57,0	63,3	43	63,3	60,0	80	60,0	55,9	39	55,9
24-30sec	32,8	86	32,8	36,4	43	36,4	34,4	80	34,4	31,3	39	31,3
33-60sec	51,5	86	51,5	55,7	43	55,7	53,6	80	53,6	50,3	39	50,3
63-90sec	15,0	86	15,0	16,1	43	16,1	14,2	80	14,2	13,8	39	13,8
93-120sec	9,4	86	9,5	8,9	43	8,9	7,7	78	7,8	7,0	37	7,4
2.03-4min	14,0	86	14,0	12,8	43	12,8	13,7	80	13,6	11,7	39	11,7
5-9min	3,1	82	3,3	2,6	40	2,8	3,3	72	3,6	2,8	35	3,1
10-14min	0,5	32	1,3	0,4	13	1,3	0,6	34	1,4	0,7	20	1,3
15-20min	0,1	11	1,1	0,1	4	1,0	0,2	10	1,4	0,2	7	1,1
>20	0,1	10	1,2	0,1	3	1,0	0,2	7	2,0	0,2	5	1,2
	**Walking**											
9-12sec	83,9	86	83,9	85,8	43	85,8	88,5	80	88,5	82,9	39	82,9
15-21sec	53,8	86	53,8	53,7	43	53,7	58,7	80	58,7	52,7	39	52,7
24-30sec	23,0	86	23,0	22,7	43	22,7	25,0	80	25,0	22,7	39	22,7
33-60sec	23,2	86	23,2	18,3	43	18,3	24,5	78	25,1	22,2	39	22,2
63-90sec	4,0	79	4,3	2,8	33	3,7	4,4	74	4,7	4,4	34	5,0
93-120sec	1,7	65	2,2	0,9	20	2,0	2,1	67	2,6	1,8	20	3,5
2.03-4min	2,6	69	3,2	1,5	21	3,0	3,3	69	3,8	2,2	23	3,7
5-9min	0,7	27	2,1	0,1	5	1,0	0,7	32	1,8	0,5	12	1,5
10-14min	0,1	8	1,0	0,0	1	1,0	0,2	12	1,3	0,1	2	1,0

15-20min	0,0	2	1,0	0,0	1	1,0	0,0	1	1,0	0,0	0	
>20	0,0	1	2,0	0,0	0		0,0	2	1,0	0,0	1	1,0
	**Running**											
9-12sec	12,0	76	13,5	7,7	34	9,7	14,5	73	15,9	9,8	30	12,8
15-21sec	6,0	66	7,8	2,5	24	4,5	6,8	69	7,9	4,7	20	9,1
24-30sec	1,5	42	3,0	0,7	16	1,9	1,9	52	2,9	1,4	14	3,9
33-60sec	0,8	28	2,4	0,4	9	1,8	1,0	34	2,4	0,9	10	3,7
63-90sec	0,1	5	1,2	0,0	1	1,0	0,1	10	1,0	0,1	2	1,0
93-120sec	0,0	2	1,5	0,0	0		0,1	7	1,1	0,0	0	
2.03-4min	0,0	1	1,0	0	0		0,1	5	1,6	0,0	0	
5-9min	0,0	0		0	0		0,0	3	1,0	0,0	0	
10-14min	0,0	0		0	0		0,0	0		0,0	0	
15-20min	0,0	0		0	0		0,0	0		0,0	0	
>20	0,0	0		0	0		0,0	0		0,0	0	

Mean All = the mean values calculated on all participants that has available data, represented by (n=). Number of observations = the number of measurement days that actually have the bout length, shown in Figure 2. mean from observations= the mean among these observations, shown in figure 3. Sec= seconds, min=minutes.
